# Senggani fruit (*Melastoma malabathricum* Linn.) extract as a natural indicator in pH-responsive PVA-taro starch plastic packaging

**DOI:** 10.55730/1300-0527.3672

**Published:** 2024-01-15

**Authors:** Rika Risma DEWI, Intan SYAHBANU, Winda RAHMALIA

**Affiliations:** Department of Chemistry, Faculty Mathematics and Natural Science, Tanjungpura University, Pontianak, Indonesia

**Keywords:** Intelligent packaging, senggani fruit, *Melastoma malabathricum* Linn

## Abstract

Polyvinyl alcohol (PVA)-starch-based bioplastics are widely used in many applications. pH-responsive plastic packaging was produced through the incorporation of senggani (*Melastoma malabathricum* Linn.) fruit extract into PVA-taro starch-based plastic packaging. The objective of this research was to examine the characteristics of senggani fruit extract under different pH conditions and explore its application as a pH indicator in intelligent packaging. The senggani fruit was extracted through the maceration method using a solvent comprising 96% ethanol and 3% citric acid, with a ratio of 85:15 (v/v). The senggani fruit extract solution underwent color changes, appearing pink at pH levels below 6, pale purple at pH 7–11, and brownish-yellow at pH 12–14. Notably, the color of the senggani fruit extract solution remained stable at pH < 5. Before the addition of the senggani fruit extract, the PVA-taro starch solution produced a brownish-yellow plastic packaging. However, following the addition of senggani fruit extract, the plastic packaging turned pink. The addition of senggani fruit extract affected the mechanical properties of plastic packaging, resulting in a reduction in swelling from 103.679 ± 2.456% to 57.827 ± 3.563%, a decrease in tensile strength value from 3.827 ± 0.603 Mpa to 1.991 ± 0.460 Mpa, and a decline in the percent elongation value from 156.250 ± 12.392% to 116 ± 6.722%. Plastic packaging incorporating senggani fruit extract exhibits color changes across the pH range of 1–14, accompanied by varying color parameter values (L, a, b, E, and WI). Therefore, it has the potential to be used as intelligent packaging for monitoring food freshness and quality.

## 1. Introduction

Packaging plays a crucial role in ensuring the safety and quality of food products. While plastic packaging made from synthetic polymers is commonly used, it poses challenges due to its non-decomposable nature in the short term. The annual high utilization of plastic leads to the accumulation of plastic waste, resulting in environmental issues. To address these challenges, various types of plastic packaging have been developed using biodegradable materials such as starch, cellulose, wheat gluten, polylactide (PLA), and polyhydroxyalkanoate (PHA) [1].

Indonesia boasts abundant biological and natural resources, especially agricultural products. Among these resources are potential candidates for the development of biodegradable plastics, including corn, cassava [1] taro, wheat, potato, and tapioca [2]. Taro stands out as a promising raw material for plastic production due to its starch content, comprising amylose and amylopectin homopolymers. The amylopectin in taro exhibits a short and branched chain structure, which can undergo elongation with the application of heat, resulting in longer average chain lengths. Temperature plays an important role in bioplastic preparation. Starch exhibits a free-flowing character above its glass transition temperature (Tg). Taro starch has a Tg between 75 °C and 80 °C. To achieve gelation, the temperature should be maintained slightly above the Tg, resulting in lower cohesive forces and increased free volume in the bioplastic solution. Therefore, the formation of bioplastic will be conducted at this temperature. [3].

In response to consumer demands for food quality and safety, packaging innovation has advanced significantly. Intelligent packaging stands out as one such innovation, not only offering information about the expiration date of food but also directly indicating food quality through various indicators [4]. These indicators typically fall into two categories: biological indicators and synthetic indicators. Commercially available synthetic indicators tend to be more expensive. Additionally, their utilization may contribute to environmental pollution, and if incorporated into food, they could potentially pose risks to human health [5]. Natural indicators offer a cost-effective and environmentally friendly alternative, presenting significant potential for further development. Commonly utilized indicators on product packaging include those monitoring changes in pH [6], O_2_, CO_2_, humidity, and temperature [5].

Generally derived from fruits and vegetables, anthocyanins are phenolic compounds that give red, purple, and blue colors. They can be used as pH indicators because they undergo structural changes and show color variations at different pH conditions [7]. Senggani (*Melastoma malabathricum* Linn.) is a plant that contains anthocyanins. Previous research has demonstrated that the extract derived from senggani fruit exhibits pronounced color changes in response to pH variations [8]. Senggani is easily found in West Kalimantan as it grows abundantly in the wild, typically found in bushes and forests.

Recently, the natural indicators incorporated with bioplastic for pH-responsive materials have attracted growing interest. The utilization of PVA-starch-based materials is particularly intriguing due to their low cost and biodegradability. Starch-PVA composites with ZnO and jamun extract as the additives have demonstrated promising responses to changes in pH [9]. Another anthocyanin source, red cabbage has successfully acted as an active and intelligent packaging material within a starch-PVA matrix, with propolis as the additive [10]. The mechanical properties of the matrix are critical factors for packaging materials. According to [11], the mechanical properties of PVA were influenced by the presence of starch due to its inherently brittle nature. To improve the mechanical properties, the inclusion of a crosslinking agent is crucial. Glutaraldehyde stands out as a common crosslinking agent for PVA-starch [12]. Another low-cost option is citric acid, which has the capability to bridge the backbone of starch and PVA [13–14].

Numerous factors affect the environmental pH of food. Monitoring changes in pH can provides an effective method for detecting food spoilage [15]. Anthocyanins can be used to detect food spoilage through their responsiveness to changes in pH. Sitanggang et al. used anthocyanin extract in gelatin as a pH indicator for smart packaging [4]. Therefore, this study aimed to enhance plastic packaging made of PVA-taro starch by incorporating anthocyanin extract as a responsive indicator, with the potential for smart packaging development.

Starch as the matrix component has garnered significant attention for its biodegradability, food safety, low cost, and ease of preparation. Taro tubers serve as a natural source of starch, with various applications in biodegradable plastic. Shanmathy et al. introduced bentonite as the reinforced taro starch biodegradable matrix. Additionally, taro tuber starch has shown promising performance when combined with chitosan [16]. The PVA-taro starch bioplastics exhibited a biodegradability of up to 65% for 8 weeks under aquatic conditions [17–18]. Senggani extract finds diverse applications, including use as food additives and colorants, in the pharmaceutical industry, and for coatings [19]. Due to the anthocyanin content of senggani fruit extract, it demonstrates potential as both a pH indicator [20] and for detecting shrimp freshness [21]. The development of pH-responsive materials for certain applications has not progressed adequately. The novelty in this research lies in the incorporation of senggani fruit extract with PVA-taro starch to create a pH-responsive material specifically designed to monitor the freshness of fish.

## 2. Materials and methods

### 2.1. Materials

The materials used in this study were hydrochloric acid (HCl) p.a, citric acid (C_6_H_8_O_7_.H_2_O) p.a, disodium phosphate heptahydrate (Na_2_HPO_4_) p.a, glycerol (C_3_H_8_O_3_) p.a, potassium chloride (KCl) p.a, sodium hydroxide (NaOH) p.a, sodium bicarbonate (NaHCO_3_) p.a, all purchased from Merck. Polyvinyl alcohol (PVA) was purchased from Sigma Aldrich. All of the chemicals used were of analytical grade. Ethanol (C_2_H_6_O) 96% used for pigment extraction was of technical grade. Senggani fruit (*Melastoma malabathricum* Linn.), the pigment source, was collected from Ayani Street, Pontianak City, and taro, the starch source, was purchased from Flamboyan Market Pontianak, Indonesia.

### 2.2. Extraction of senggani fruit

The senggani fruit was collected at Ayani Street, Pontianak City. After removing dirt and rinds, it was weighed at 600 g and then mashed with a blender. Extraction was carried out through maceration for 3 cycles of 24 h. The solvent was replaced every 24 h with an ethanol solution consisting of 96% ethanol and 3% citric acid in a ratio of 85:15 (v/v) until all samples were fully submerged. The filtrate was collected and evaporated using a rotary evaporator. The resulting extract was obtained after a vacuum drying treatment.

### 2.3. pH sensitivity test on senggani fruit extract

Senggani fruit extract was weighed at 125 mg and dissolved in 25 mL of 96% ethanol and 3% citric acid with a ratio of 85:15 (v/v). Subsequently, 1 mL of extract solution was dispensed into 14 test tubes, followed by the addition of 5 mL of buffer solution with a pH range of 1–14 [22]. The preparation of the buffer solution with a pH range of 1–14 is described below. Color changes were observed and the maximum wavelength spectrum was measured using a UV-Vis spectrophotometer (Shimadzu UV 2600) in the wavelength range of 400–800 nm [23].

A buffer solution of pH of 1 was prepared by mixing 100 mL KCl 0.02 M and HCl 2 N until a pH of 1 ± 0.1 was obtained [24]. For a buffer solution with a pH of 2, 25 mL of KCl 0.2 M and HCl 0.2 M were mixed until a pH of 2 ± 0.1 was reached. Buffer solutions with pH values ranging from 3 to 8 were prepared using citric acid solution 0.1 M and Na_2_HPO_4_ solution 0.2 M, adjusting the volumes as necessary to achieve the desired pH. Buffer solutions with pH values of 9 to 10 were prepared by mixing NaHCO_3_ 0.05 M and Na_2_CO_3_ solution. Buffer solutions with pH values of 11 and 12 were obtained by mixing 50 mL of Na_2_HPO_4_ 0.05 M with NaOH 1 M. Buffer solutions with pH values of 13 were prepared by mixing 25 mL of KCl 0.2 M with NaOH 0.2 M until a pH value of 13 was reached [25]. Lastly, buffer solutions with a pH value of 14 were obtained by NaOH 1 M solution [26]. All processes were monitored using a pH meter (Mettler Toledo S220 basic).

### 2.4. Taro starch extraction

The taro was peeled, washed thoroughly, and weighed 825 g and then blended with water in a ratio of 1:2 (w/v). The resulting mixture was filtered using a cloth. Subsequently, the pulp was added to 1 L of water, blended again, and filtered once more. The two liquids were combined and left to settle for 24 hours to facilitate the separation and decanting of the liquid and starch residue. Afterwards, the mixture was further mashed with a blender and sieved through a 100-mesh sieve. The starch precipitate obtained was dried under sunlight.

### 2.5. Preparation of plastic packaging made of PVA-taro starch with senggani fruit extract

The bioplastics was prepared using the slip casting method. Initially, 2.5 g of PVA was weighed and dissolved in 25 mL of distilled water in a beaker, heated at 90 °C with a magnetic stirrer until fully dissolved. Simultaneously, 2.5 g of taro starch, 1.25 g of citric acid, and 1 mL of glycerol were dissolved in 25 mL of distilled water in another beaker. After the PVA was dissolved, a solution containing starch, citric acid, and glycerol dissolved in distilled water was added. The mixture was then heated at 90 °C for 10 min and stirred with a magnetic stirrer for 50–60 min until a viscous solution was obtained. The same procedure was carried out with the addition of senggani fruit extract. Referring to [27], 1 g of Senggani fruit extract was weighed and dissolved in 10 mL of 96% ethanol and 3% citric acid in a ratio of 85:15 (v/v). The diluted extract, up to 2.5 mL, was pipetted into a cold bioplastic solution and stirred using a magnetic stirrer. Subsequently, the mixture was printed onto a 15 cm × 21 cm acrylic plate and allowed to stand for 2 × 24 h at room temperature (28 °C). After drying, the bioplastic was removed from the acrylic plate and tested.

### 2.6. Characterization of plastic packaging made of PVA-taro starch with senggani fruit extract

Characterization of the PVA-taro Starch involved conducting pH sensitivity test, water resistance test, assessing mechanical properties, and evaluating the suitability of the bioplastic for food packaging applications. All characterization processes are described below.

#### 2.6.1. pH sensitivity test of plastic packaging made of PVA-taro starch

The plastic packaging made of PVA-taro starch, both with and without the addition of senggani fruit extract, was cut into pieces measuring 2 cm × 1.5 cm. Subsequently, these pieces were placed into separate Petri dishes containing buffer solutions with a pH range of 1–14, and any observed color changes were noted. The samples were then positioned on white paper as a background [28]. The color measurements were conducted using a colorimeter (Linshang LS170), yielding the following values: L* = 93.698, a* = 1.123 and b* = −5.310. These values represent the color changes observed, with L measuring brightness, and a and b representing color variations. Additionally, the color difference (ΔE) and whiteness index (WI) values were calculated using the following formula [29]:


(1) $$\Delta E=\sqrt{{\left(L*-L\right)}^{2}+{\left(a*-a\right)}^{2}+{\left(b*-b\right)}^{2}}$$

(2) $$\text{WI}=100-\sqrt{{\left(100-\text{L}\right)}^{2}+{\text{a}}^{2}+{\text{b}}^{2}}$$

#### 2.6.2. Water resistance test

The test for swelling involved weighing the bioplastic, cut to a size of 2.5 cm × 2.5 cm, and placing into a container containing 20 mL of distilled water for 1 h. Subsequently, the bioplastic samples were removed from the water, reweighed, and the water resistance was calculated [28]. The water resistance test was repeated three times.

#### 2.6.3. Mechanical properties

The mechanical properties of the bioplastic were tested by preparing a sample measuring 5 cm x 1 cm for tensile strength and elongation using the Universal Testing Machine at a speed of 1 mm/s. The data obtained were then used to calculate the value of tensile strength and elongation.

#### 2.6.4. Application of plastic packaging on freshness of fish

Tuna, cleaned and weighing 25 grams, was placed into a plastic container. The container was then placed in the refrigerator with storage times for varying durations: 24 h, 48 h, 72 h, 96 h, and 120 h. Color changes in the plastic packaging were observed during this period. Subsequently, the plastic packaging made of PVA-taro starch, both with and without the addition of senggani fruit extract, was cut into pieces measuring 3 cm × 2 cm. These pieces were then placed on top of the fish, and the container was closed.

### 3. Results and discussion

For the application as a pH-sensitive material, here we evaluate the characteristics of senggani fruit extract, including the properties at different pH conditions. This is followed by a study of physical and mechanical properties study when the pigment is incorporated into PVA-taro starch matrix. PVA-taro starch without the addition of senggani fruit extract addition was used as a reference. The performance of pH-responsive materials was then observed in fish packaging.

### 3.1. Characteristics of senggani fruit extract

**Figure 1:**
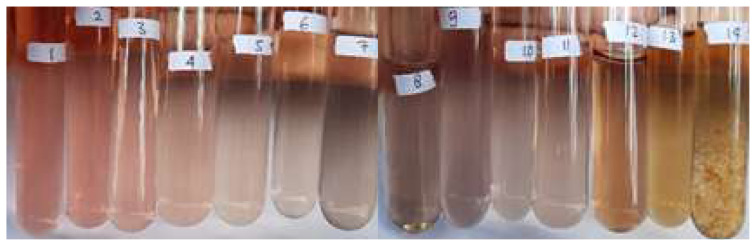
Changes in the color of the senggani fruit extract solution at (a) pH 1–7 and (b) pH 8–14.

Senggani fruit extract was successfully obtained with ethanol:citric acid 3% (85:15) as the solvent. The addition of citric acid to the solvent aimed to maintain conditions necessary to prevent the degradation of anthocyanin in the extract. The anthocyanin extract from Vietnamese *Carissa carandas* L. fruit exhibited good color stability and less anthocyanin degradation when citric acid was added [30]. Extraction of *Melastoma malabathricum* fruit using acidified ethanolic solvent gave high mean anthocyanin yields of 880.923 mg/100 g [19]. Similarly, the extraction of *Hibiscus subdariffa* with acidified ethanol (85:15) showed that the acidic condition of the solvent increased the efficiency of anthocyanin extraction. It can be concluded that the addition of acid to the extraction medium significantly stabilized anthocyanins, leading to increased extraction efficiency. Acidic conditions provide a favorable environment for the formation of flavylium chloride salts from simple anthocyanins and improve the efficiency of anthocyanin extraction [31]. Senggani fruit extract exhibited a color change across the pH range of 1–14, as depicted in Figure 1. At pH 1–3, the extract appeared pink and gradually faded at at pH 4–6. The senggani fruit extract displayed a pale purple color at pH 7–11, slowly turning brownish-yellow from pH 12 to pH 14, ultimately forming a precipitate. Interaction of the senggani fruit extract solution with high pH (alkaline) solutions led to instability, resulting in precipitation, indicating the solution’s instability.

The formation of the precipitate can occur due to aggregation, which is a process where particles approach each other, collide, and settle within the solution [32].

**Figure 2:**
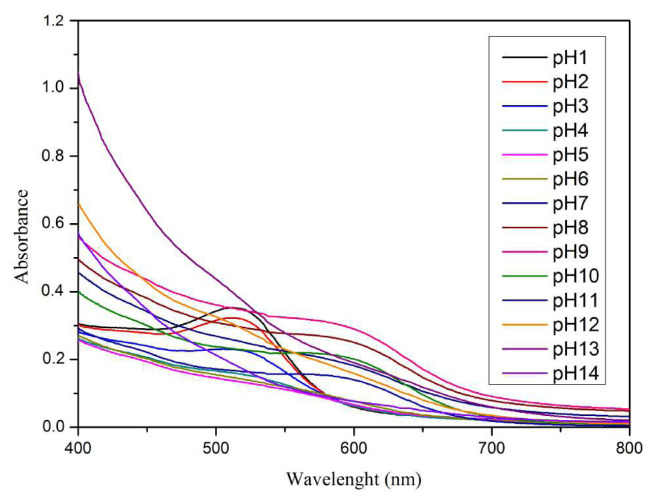
UV-Vis spectrum of senggani fruit extract at pH 1–14.

The color change of the Senggani fruit extract solution at various pH shows the absorption peaks of different wavelengths, as depicted in Figure 2. At pH 1–8, the peak of the maximum absorption wavelength increased (bathochromic shift) and decreased the absorbance value to pH 6. At pH 7 and 8, the absorbance value increased. At pH 9, the maximum wavelength decreased to pH 11, along with a decrease in the absorbance value. At pH 12, the maximum wavelength increased with an increase in the absorbance value. At pH 13 and pH 14, there was no maximum wavelength spectrum.

Changes in pH can cause structural alterations in the anthocyanin compound molecules that cause color variations. In acidic solutions, the anthocyanin structure changes to flavylium cation (red) and quinoidal anhydrase (purple). In alkaline solutions, it changes to chalcone (yellow) [29]. The anthocyanin structures at pH 1–3 appeared as flavylium cations, while at pH 4–5, they presented as carbinol pseudobases. At pH 6–7, anthocyanin had a quinoidal base structure; however, at pH 7–8, it presented as an anionic quinoidal base. At pH 9, anthocyanin had a chalcone structure [33].

**Figure 3:**
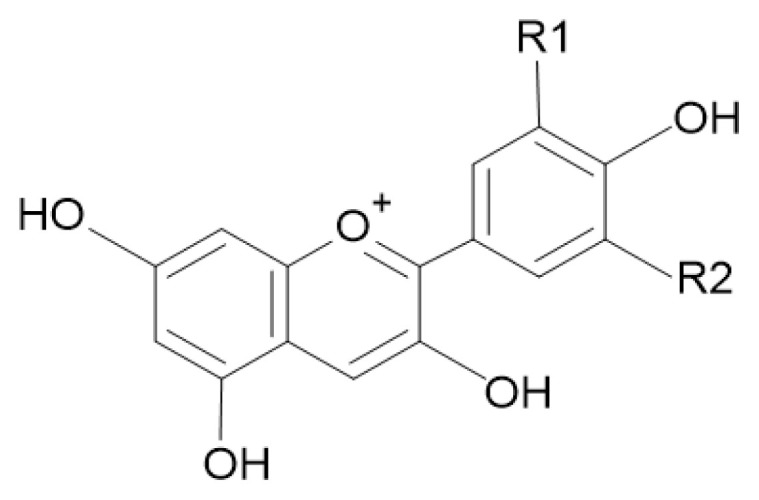
Anthocyanidin structure: (a) cyanidin (R1 = −OH; R2 = −H), (b) delphinidin (R1 = −OH; R2 = −OH) [32].

Phytochemical screening showed that *Melastoma malabathricum* exhibited a strong response to presented flavonoids and phenolics [34]. Anthocyanin remains the major component in *Melastoma malabathricum* fruit extract. Identification of anthocyanins using UPLC-ESI-MS/MS revealed that *Melastoma malabathricum* fruit extract contains cyanidin and delphinidin compounds [19]. Figure 3 illustrates the structures of cyanidin and delphinidin [35].

**Figure 4:**
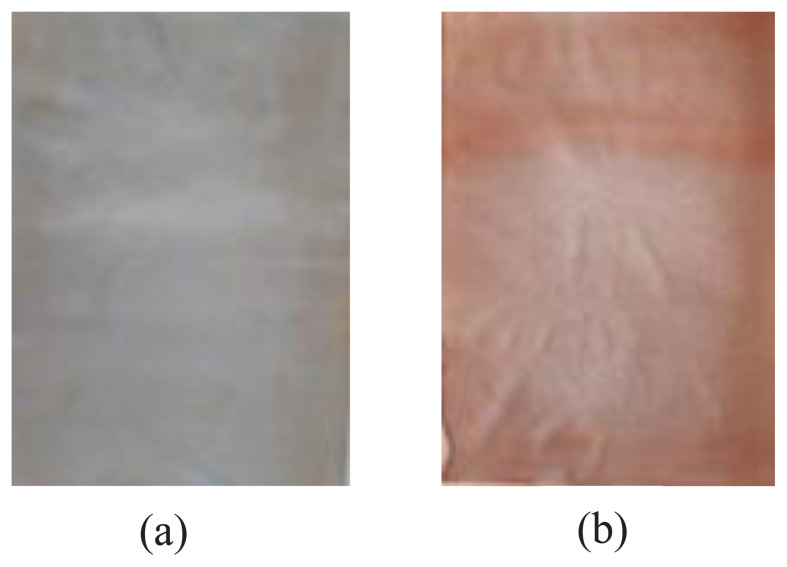
PVA plastic packaging-taro starch (a) without senggani fruit extract, (b) with the addition of senggani fruit extract.

Plastic packaging made from PVA-taro starch produced both with and without the addition of senggani fruit extract yielded brownish-yellow and pink plastic, respectively, as depicted in Figure 4.

**Figure 5:**
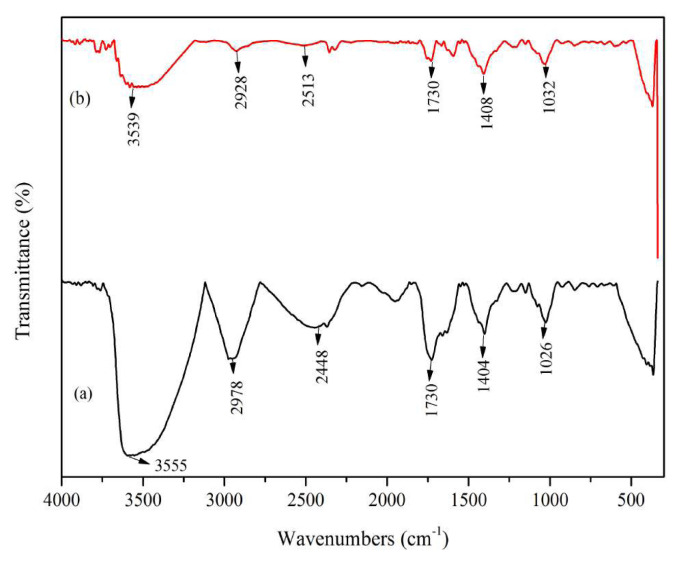
The spectrum of plastic packaging made of PVA-starch (a) without the addition of senggani fruit extract and (b) with the addition of senggani fruit extract.

The IR spectrum depicted in Figure 5 was generated from plastic packaging fabricated from PVA-taro starch, both with and without the inclusion of senggani fruit extract.

The figure shows a shift in the absorption band between the functional groups with and without the addition of senggani fruit extract that occurs due to the interaction between Senggani fruit extract and PVA-starch. The absorption peaks at wave numbers 3555 cm^−1^ and 3539 cm^−1^ represent the OH stretching vibration. The wavenumbers 2978 cm^−1^ and 2928 cm^−1^ indicate the absorption peak of the CH asymmetric stretching vibration [4]. The presence of C=O stretching is indicated by the peaks at 1730 cm^−1^ and 1755 cm^−1^ [13]. Additionally, the absorptions at 1404 cm^−1^ and 1408 cm^−1^ indicate the CH-CH_2_ vibration [36]. Furthermore, peaks observed at wave numbers 1026 cm^−1^ and 1032 cm^−1^ show the absorption peak of the C-O-C stretching vibration [13].

There were no significant shifts in absorption between groups with or without senggani fruit extract. However, changes in intensity were observed in peak absorption at 3539 cm^−1^ and 1730 cm^−1^. The peak intensity of hydroxyl groups and C=O groups of PVA-taro starch with senggani fruit extract addition remains lower than the other one. This decreased absorption intensity might be caused by the functional groups of organic compounds in senggani fruit extract that had reacted with hydroxyl groups of PVA and starch and were involved in crosslinking reactions in the presence of citric acid. The formation of hydrogen bonding involves the OH groups of the PVA, starch, and senggani fruit extract with the COOH groups of citric acid [13].

### 3.2. Physical characteristics of the plastic packaging

The mechanical properties of the plastic packaging made from PVA-taro starch are presented in Table 1. The addition of Senggani fruit extract to plastic packaging lowered both tensile strength and elongation values. The addition of fruit extract into the film leads to a decrease in tensile strength, thereby altering its mechanical properties [37]. Senggani fruit extract contains numerous organic compounds with major components from the anthocyanin groups. Table 1 shows the swelling percentage of the sample after the addition of senggani fruit extract. When the organic compound is incorporated, there are two possibilities. First, at lower concentrations of anthocyanin, the mechanical properties of the bioplastic could be improved. On the contrary, another study showed that at higher concentrations, the mechanical properties decreased due to the unstabilized polymeric network by excessive anthocyanin [36]. This phenomenon decreased both mechanical properties—the tensile strength and the percentage of elongation—in this research.

The swelling value for PVA-starch with Senggani fruit extract decreased due to the presence of additives thus restricting the water penetration into the bioplastic [38]. This restriction was confirmed by FTIR data (Figure 5), which showed a decreased intensity of the hydroxyl group after the incorporation of senggani fruit extract lowered swelling capacity. The decreased intensity of hydroxyl groups reduces the interaction of bioplastic with water [23]. Moreover, the senggani fruit extract used in this research was a crude extract containing waxy and resinous components, which contributed to the formation of hydrophobic surfaces and led to a lower swelling capacity [36]. Swelling capacity influences the biodegradability of bioplastic. Plastics with a lower percentage of swelling capacity are better due to their ease of damage or decomposition [39].

### 3.3. pH-responsive plastic packaging

**Figure 6:**
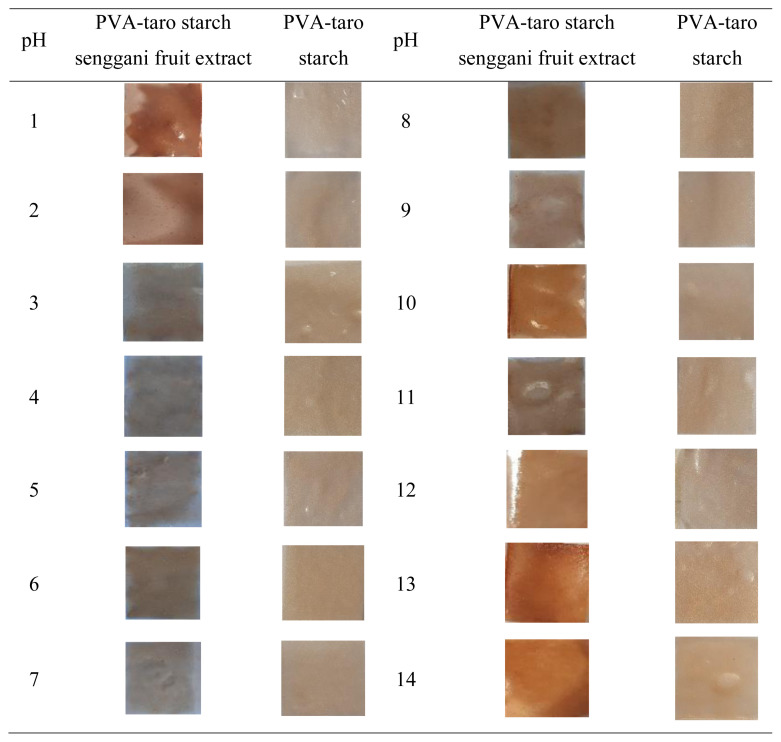
Changes in the color of plastic packaging at pH 1–14.

The color response of plastic packaging made from PVA-taro starch in the pH range of 1–14 is depicted in Figure 6. At pH 1, the plastic packaging appears pink and gradually fades until pH 5. At pH 6, it exhibits an orange color change. Furthermore, at pH 7, it appears brownish yellow, and as the pH increases to 14, the color of the plastic packaging shifts towards brown.

Color changes in plastic packaging made from PVA-taro starch with the addition of senggani fruit extract result from changes in the anthocyanin structure at different pH levels. The color change comes from flavylium cations under acidic conditions turning into quinoidal anions under alkaline conditions [29]. Meanwhile, in plastic packaging without the addition of Senggani fruit extract, no color change occurs.

The color parameters L and WI values ranging from 0–50 indicate a dark-colored film, while those from 51 to 100 signify a light color. In this context, a positive value (a +) indicates red, a negative (a −) represents green, b positive (b +) means yellow, and b negative (b −) denotes blue [29]. Table 2 shows the values of L and WI, where brightness levels that tend to decrease with increasing pH. A positive value indicates a reddish color, which decreases until pH 7, increases from pH 8 to 12, and then decreases again until pH 14. Similarly, a positive b value indicates a yellow color at pH > 8 with a value of b greater than pH < 8. The WI value, which tends to decrease with increasing pH, indicates that the plastic packaging is getting darker. The total color difference value (Δ E) shows the difference from plastic packaging, which decreases until pH 7 and then increases from pH 8 to 14. These color parameters indicate that plastic packaging with the addition of senggani fruit extract exhibits a color change response to variations in pH.

**Figure 7:**
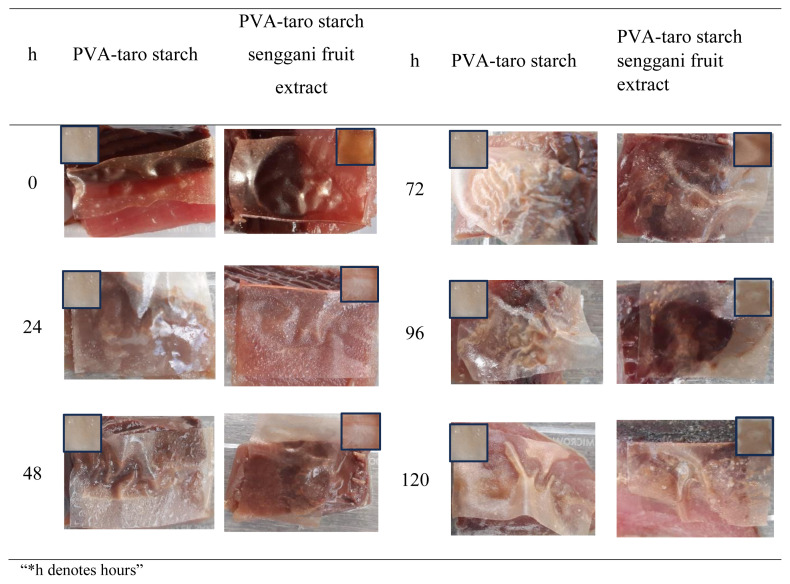
Changes in the color of plastic packaging with time variations.

The PVA-taro starch with senggani fruit extract was applied to observe its performance in evaluating fish spoilage at chiller temperature (approximately 5 °C). Figure 7 illustrates the applications of the bioplastics. Initially, it displayed a pink color, which faded to pale pink after 24 h. Subsequently, a clear brownish color of the bioplastic was observed after 48 h, coinciding with the fish reaching a pH of 8. Additionally, physical examination of the fish revealed signs of spoilage beginning after 48 hours. On the other hand, the plastic packaging made of PVA-taro starch without the addition of senggani fruit extract exhibited no color change from the beginning up to 120 h of storage. This phenomenon suggests that the incorporation of senggani fruit extract into PVA-taro starch bioplastic responds to changes in the condition of the fish, indicating its potential as an intelligent packaging material.

The color changes observed suggest that plastic packaging with the addition of senggani fruit extract is responsive to alkaline compounds emitted from fish spoilage. These compounds are produced by bacterial activity during protein decomposition. This activity produces alkaline and volatile nitrogen (Total Volatile Bases), influencing the increase in pH and foul odor [9,40]. Plastic packaging containing senggani fruit extract demonstrates a fast response to Total Volatile Bases (TVB) [23], enabling real-time monitoring of fish freshness during storage.

## 4. Conclusion

The PVA-taro starch was prepared both with and without the addition of senggani fruit extract. Senggani fruit extract exhibited varying responses to different pH conditions (ranging from pH 1 to 14) due to the presence of anthocyanin. The FTIR data showed that the addition of senggani fruit extract did not cause a shift in the wavenumber. However, there were reduced peak intensities observed at 3539 cm^−1^ and 1730 cm^−1^. This phenomenon might be caused by hydrogen interactions present in the bioplastic. As a result, both mechanical properties and swelling degree were reduced for the PVA-taro starch with senggani fruit extract addition. A study conducted on PVA-taro starch in different pH conditions revealed that pigment addition contributed to the color change of the bioplastic. Additionally, it exhibited a response when tested on fish products. Therefore, it demonstrates potential as intelligent packaging for monitoring the freshness and quality of food, especially fish products.

## Figures and Tables

**Table 1:** Mechanical properties of plastic packaging.

Sample	Tensile strength (Mpa)	Elongation (%)	Swelling (%)
PVA-taro starch	3.827 ± 0.603	156.250 ± 12.392	103.679 ± 2.456
PVA-taro starch Senggani fruit extract	1.991 ± 0.460	116 ± 6.722	57.827 ± 3.563

Information: Data is presented in the form of mean ± standard deviation.

**Table 2:** Color parameters of plastic packaging PVA-taro starch with the addition of senggani fruit extract.

pH	L	a	b	ΔE	WI
1	72.463 ± 1.255	17.683 ± 0.681	25.209 ± 0.370	40.742 ± 1.227	58.646 ± 1.380
2	71.281 ± 2.690	11.915 ± 1.970	22.701 ± 3.544	37.473 ± 4.803	61.482 ± 4.672
3	76.583 ± 3.532	11.521 ± 1.818	20.459 ± 1.197	32.740 ± 3.213	66.785 ± 3.729
4	77.755 ± 1.921	10.497 ± 1.004	13.927 ± 3.820	26.867 ± 4.181	71.466 ± 3.707
5	74.573 ± 1.276	11.293 ± 0.531	19.704 ± 2.232	33.335 ± 1.030	65.520 ± 0.721
6	75.511 ± 1.575	9.227 ± 1.310	21.924 ± 5.606	33.817 ± 5.636	65.719 ± 4.973
7	78.052 ± 0.788	7.687 ± 0.477	15.482 ± 0.939	26.857 ± 1.200	72.047 ± 1.143
8	76.717 ± 5.868	9.233 ± 3.461	17.879 ± 8.753	29.884 ± 11.057	69.065 ± 10.411
9	69.797 ± 0.561	9.119 ± 0.347	18.615 ± 1.349	34.759 ± 1.359	63.354 ± 1.201
10	65.816 ± 1.737	11.191 ± 1.129	26.163 ± 1.763	43.250 ± 2.569	55.508 ± 2.549
11	69.035 ± 1.159	8.294 ± 1.456	19.525 ± 2.999	35.822 ± 2.433	62.362 ± 2.050
12	51.907 ± 2.440	19.512 ± 1.652	34.662 ± 4.800	60.732 ± 4.982	37.526 ± 4.692
13	62.175 ± 5.124	11.167 ± 2.128	27.533 ± 0.994	46.815 ± 3.364	51.751 ± 4.061
14	56.684 ± 4.385	17.368 ± 2.059	39.776 ± 1.728	60.679 ± 2.167	38.570 ± 2.736
